# Mapping the path to degeneration: How lesion location predicts post-stroke cerebellar atrophy

**DOI:** 10.1016/j.nicl.2025.103875

**Published:** 2025-08-30

**Authors:** Makayla Gibson, Roger Newman-Norlund, Janina Wilmskoetter, Sigfus Kristinsson, Leonardo Bonilha, Julius Fridriksson, Chris Rorden

**Affiliations:** a Department of Psychology, University of South Carolina, Columbia, SC, USA; b Division of Speech-Language Pathology, College of Health Professions, Medical University of South Carolina, Charleston, SC, USA; c Department of Neurology, University of South Carolina School of Medicine, Columbia, SC, USA; d Department of Communication Sciences & Disorders, University of South Carolina, Columbia, SC, USA

**Keywords:** Cerebellum, Stroke, Magnetic resonance imaging, Speech disorders, LSM

## Abstract

**Introduction:**

Stroke can lead to neurological changes beyond the initial lesion site, including post-stroke crossed-cerebellar degeneration. While traditional methods typically rely on total lesion volume to assess remote effects, the spatial distribution of lesions may more accurately predict cerebellar atrophy and associated functional deficits. This study investigated whether anatomically specific cortical lesions contribute to cerebellar gray matter volume loss, expanding on the hypothesis that cerebellar atrophy may reflect more than global brain injury severity, and instead result from targeted disruption of cortico-cerebellar pathways.

**Methods:**

Data from 142 chronic left hemisphere stroke participants (57 females, 85 males; *M =* 61 years) were analyzed using voxel-based lesion-symptom mapping (VLSM) and region of interest (ROI) analyses. High-resolution structural MRI scans were processed to quantify cerebellar gray matter volume (GMV) across 28 spatially distinct regions, with lesions manually delineated and mapped.

**Results:**

Significant associations were found between lesion load in specific white matter tracts and cerebellar GMV atrophy. Lesions in the posterior limb of the internal capsule, retrolenticular part of the internal capsule, fornix/stria terminalis and superior corona radiata were strongly linked to GMV reductions in right cerebellar lobules and vermis. Notably, cortical lesions primarily impacted the right cerebellar regions, supporting the concept of post-stroke crossed cerebellar diaschisis. In a complementary analysis, voxel-based lesion-symptom mapping using Apraxia of Speech (AOS) severity scores revealed overlapping lesion predictors with those driving cerebellar degeneration, particularly within the superior corona radiata and retrolenticular part of the internal capsule. This overlap suggests that damage to these tracts may contribute jointly to speech-motor impairment and remote cerebellar atrophy.

**Conclusion:**

The spatial distribution of cortical lesions, rather than total lesion volume, plays a crucial role in predicting cerebellar degeneration. These findings suggest the importance of considering lesion location in stroke rehabilitation, and provide further insights into the functional connections between the cerebrum and cerebellum.

## Highlights

• Lesion location predicts cerebellar atrophy beyond total lesion volume.
• Key white matter tracts (PLIC, SCR, RLIC, Fx/ST) linked to cerebellar GMV loss.
• Right cerebellar lobules degenerate after left-hemisphere cortical stroke.
• SCR and RLIC lesions predict both AOS severity and cerebellar degeneration.
• Lesion-atrophy links support a network-based model of post-stroke outcomes.

## 1 Introduction

Stroke remains a leading cause of long-term disability worldwide (Katan and Luft, 2018), leading to neurological changes that can manifest in brain regions beyond the initial lesion site. Among these remote effects, post-stroke cerebellar degeneration represents a significant but often overlooked phenomenon with substantial implications for functional recovery. When cerebral lesions occur, they can trigger crossed cerebellar diaschisis (CCD), characterized by reduced metabolism, blood flow, and eventual structural degeneration in the cerebellar hemisphere contralateral to the cortical lesion (Infeld et al., 1995). This remote degeneration reflects the disruption of critical cortico-cerebellar pathways that would normally maintain cerebellar integrity and function.

These disrupted pathways provide a theoretical framework for understanding motor speech disorders, such as apraxia of speech (AOS), emphasizing the potential relationships between cortical and cerebellar motor systems. Given the extensive connectivity between the cortex and cerebellum, damage to the left hemisphere cortical speech networks, particularly in motor and somatosensory regions such as the precentral gyrus, postcentral gyrus, superior corona radiata, superior longitudinal fasciculus, and supramarginal gyrus, has been associated with AOS and may result in broader network-level disruptions (Basilakos et al., 2015). The cerebellum’s established role in motor sequencing provides a strong theoretical basis for understanding how impairments in the cortico-cerebellar pathway may impact speech motor functions, particularly the timing and coordination of articulatory movements (Lundin, 2023, Durisko and Fiez, 2010). Although investigations linking cerebellar changes to AOS are limited, the structural and functional connections between these regions highlight the need for further investigation into the neural mechanisms. These connections not only inform models of the intact human brain but also hold translational relevance for rehabilitation. Notably, recent work suggests that cerebellar stimulation may enhance aphasia treatment outcomes (Sebastian, 2020).

Diaschisis, the process in which a lesion in one area causes dysfunction in a remote but connected region, is well-documented following stroke (Carrera and Tononi, 2014). While CCD often resolves spontaneously, it can persist chronically in some individuals (Nocuń et al., 2013) and may underlie chronic crossed cerebellar degeneration observed in diffusion imaging studies (Kohannim et al., 2018). However, the mechanisms underlying diaschisis, particularly CCD, remain incompletely understood. One explanation could be that cerebellar degeneration reflects just the overall lesion burden, with greater global damage leading to greater secondary atrophy. Alternatively, disruption to key white matter tracts involved in cortico-cerebellar communication may produce specific focal cerebellar degeneration. At the foundation of this idea, task-based functional magnetic resonance imaging (fMRI) in healthy individuals have revealed distinct cerebellar activation patterns that form reproducible functional atlases (Nettekoven, 2024, Stoodley and Schmahmann, 2009); and topographic maps linking cerebellar and cerebral regions have been generated using resting-state networks (Brissenden, 2018, Buckner et al., 2011, Guell et al., 2018); These studies have painted a picture of the domain-specific functional organization of the cerebellum, showing that anterior regions support sensorimotor function, while posterior regions, specifically Crus I/II and lobule IX, are involved in language, working memory and moreso default mode processing. Because fMRI data are based on vascular behavior, rather than direct neural activity, some studies may reflect delays in hemodynamic response instead of true functional coupling.

Taken together, these findings suggest that a sufficiently large sample of chronic stroke patients may exhibit robust, spatially specific patterns of cerebellar atrophy that reflect underlying lesion location and connectivity. Identifying how chronic cerebral injuries lead to cerebellar degeneration offers a unique opportunity to map these relationships. Unlike fMRI-derived atlases, lesion-based approaches provide directional insight into how focal cortical damage reorganizes downstream cerebellar circuits. Our objective was to directly test this hypothesis and to discover the mapping between lesion location and the extent of crossed cerebellar atrophy. Establishing such a topographic relationship would not only validate and extend existing functional atlases but also yield clinically meaningful biomarkers of remote degeneration, informing prognosis and intervention strategies in stroke recovery.

More specifically, the cortico-cerebellar pathways, which play a crucial role in coordinating motor and speech functions, contain mostly contralateral projections traveling through key white matter structures, including the posterior limb of the internal capsule and corona radiata (Stoodley and Schmahmann, 2009, Silveri, 2021). These pathways, comprising corticopontine tracts and pontocerebellar fibers (Palesi, 2017); provide the anatomical substrate for CCD following a cortical or subcortical stroke. In the context of motor speech, these pathways are particularly critical, as they translate speech motor plans into precise, coordinated muscular movements (Ackermann et al., 2007). When damaged, they can lead to distinctive motor speech programming deficits characterized by difficulties in initiating and sequencing speech sounds, imprecise articulatory movements, and altered prosodic features.

Traditional approaches to understanding post-stroke outcomes have heavily relied on total lesion volume as a primary predictor (Khalilian et al., 2025, Schiemanck et al., 2006). However, this gross measure fails to capture the relationship between specific lesion locations and remote degenerative processes. This limitation is particularly evident in complex functions such as speech motor control, as mentioned above, which depends on distributed neural networks rather than isolated brain regions. The differences in recovery patterns observed clinically suggest that the spatial distribution of lesions may be more informative than just overall lesion size in predicting both cerebellar degeneration and associated functional deficits (Bates, 2003).

Despite the recognized value of cortico-cerebellar pathways (Schulz, 2017), our understanding of how damage to specific portions of these pathways impacts cerebellar integrity remains limited. This knowledge gap could be particularly significant in understanding variations in speech recovery and motor programming deficits following stroke.

In this study, we address these knowledge gaps by investigating the relationship between lesion location and cerebellar gray matter volume (GMV) degradation in chronic stroke survivors. We employ voxel-based lesion-symptom mapping (VLSM) and ROI-based analyses to identify specific cortical and subcortical regions where damaged tissue most strongly predicts cerebellar atrophy, independent of total lesion volume and age. We hypothesize that specific lesion locations will predict cerebellar GMV reduction beyond the contribution of total lesion volume, challenging the assumption that cerebellar degeneration following cortical stroke is solely a function of overall damage severity. Furthermore, we propose that lesions affecting the posterior limb of the internal capsule and the corona radiata will demonstrate the strongest relationship to cerebellar atrophy due to their role in cortico-cerebellar connectivity.

By investigating the spatial distribution of lesions and identifying those most predictive of cerebellar degeneration, this research provides a deeper understanding of how brain injury disrupts the structural integrity of remote but functionally connected regions. These findings may reveal critical disruptions in pathways supporting speech motor control, explaining the variations in speech recovery patterns observed clinically. The approach shifts our understanding from a purely localizationist view to a dynamic, network-based model of motor speech recovery.

Ultimately, identifying regions most associated with cerebellar atrophy may deepen our understanding of stroke-related network disruptions. While the present data are observational and retrospective, such associations could inform future prospective research on cerebellar vulnerability and structural disconnection in stroke recovery. By developing more nuanced predictive models of speech recovery and understanding the complex neural reorganization following stroke, we can move toward more targeted and effective intervention approaches.

## 2 Methods

### 2.1 Participants

Data for this study were drawn from the Aphasia Recovery Cohort (Gibson, 2024); which included participants recruited from multiple studies who provided informed consent under protocols approved by the Institutional Review Board at the University of South Carolina (USC) in Columbia, SC. Eligibility criteria required participants to have experienced a left-hemisphere stroke at least six months prior, be between 21 and 80 years of age, and have no contraindications to MRI or additional neurological conditions (e.g., multiple sclerosis, Parkinson’s disease, dementia) (Gibson, 2024). In this cohort, 142 unique individuals underwent scanning (57 females, 85 males; 29–80 years old, *M* = 61, *SD* = 10). On average, imaging was conducted 5.1 years post-stroke (*SD* = 4.7 years).

### 2.2 Imaging data acquisition

Structural MRI scans were obtained for all participants at the McCausland Center for Brain Imaging using a Siemens Trio/Prisma 3 T scanner with a 20-element head-neck coil. High-resolution T1-weighted images were collected using an MP-RAGE sequence with the following parameters: 1 mm isotropic voxels, 256 × 256 matrix size, 9-degree flip angle, 192 slices, repetition time of 2250 ms, inversion time of 925 ms, and echo time of 4.11 ms (Gibson, 2024).

### 2.3 Neuroimaging analysis

Cerebellar volume and total intracranial volume (TIV) were measured using high-resolution T1-weighted structural MRI images. Image processing was performed using MATLAB (MathWorks, 2022b), SPM12 (version 7781) (Penny et al., 2011); and the Cat12 (v12.6, 1700) VBM toolbox with default parameters (Gaser, xxxx). To ensure accurate spatial normalization, T1-weighted images underwent enantiomorphic healing prior to processing (Nachev et al., 2008, Yourganov et al., 2018). Cerebellar volume metrics were extracted through the standard Cat12 pipeline and adjusted for TIV to account for individual variability in brain size (Whitwell et al., 2001). This adjustment converted the regional volume values to a percentage of total brain volume, producing a normalized metric that corrects for differences in TIV across participants (Whitwell et al., 2001) Gray and white matter volumes were computed as percentages for the 28 distinct regions defined by the Spatially Unbiased Atlas Template of the human cerebellum (SUIT) (Diedrichsen et al., 2009).

Chronic stroke lesions were manually traced by trained raters using MRIcroGL12 software. Binary NIFTI lesion masks were created in native space, with lesioned voxels labeled as ‘1′ and non-lesioned voxels labeled as ‘0.’ Lesion volume was calculated by summing the lesioned voxels and converting the total to cubic millimeters based on native voxel dimensions, ensuring accurate volume quantification while preserving spatial alignment.

**Fig. 1:**
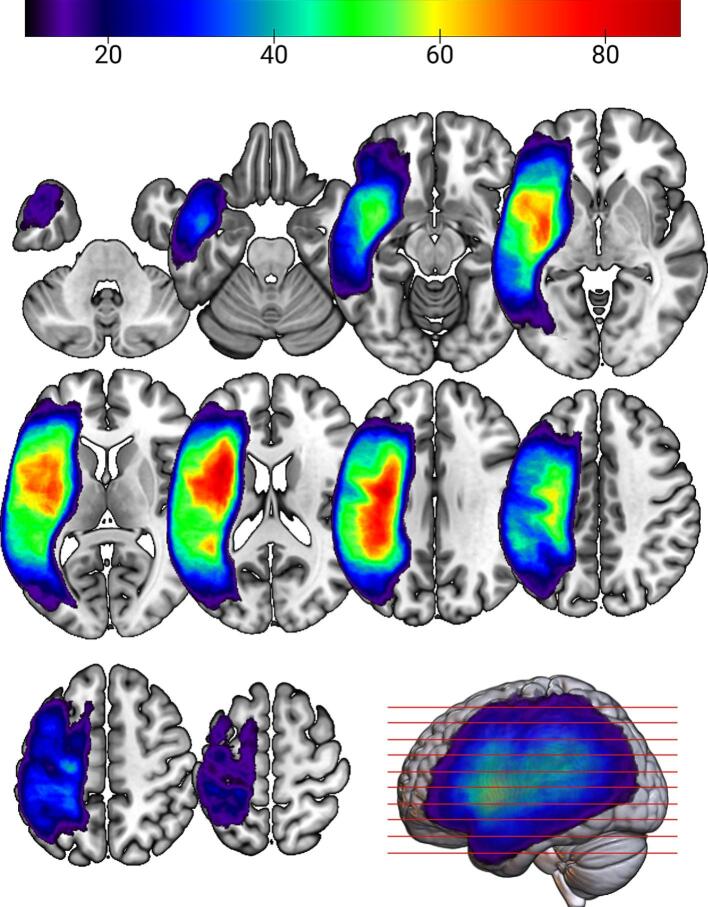
Group lesion distribution map displayed in neurological convention. The slices correspond to the MNI coordinate space at z-dimension intervals of −35, −24, −13, −3, 8, 18, 29, 39, 50, and 60. Lesion intensity is represented by a color gradient, with darker blue areas (value = 10) indicating minimal overlap and brighter red areas (value = 89) representing the most commonly affected regions, with the gradient showing overlap from 10 to 89 individuals. (For interpretation of the references to color in this figure legend, the reader is referred to the web version of this article.)

To illustrate lesion distribution across the cohort, an overlay map was generated by summing individuals’ normalized binary lesion masks. The resulting lesion map (Fig. 1) highlights regions with the highest lesion frequency, where cooler areas (value = 10) indicate minimal overlap and warmer areas (value = 89) correspond to the most commonly affected regions.

### 2.4 Lesion symptom mapping analysis

We performed univariate lesion-symptom mapping (LSM) using the NiiStat Toolbox (NIRTC); which has been previously used to analyze neuroimaging data in individuals with aphasia (Basilakos et al., 2015, Harrington, 2024, Hillis, 2018, Johnson, 2022). Our analysis included two variables (regional cerebellar GMV and age) with normalized lesion maps, compared to 85 left-hemisphere (LH) regions of interest (ROIs) from the Johns Hopkins University (JHU) grey and white matter atlas (Infeld et al., 1995); while regressing out lesion volume. Since our cohort exclusively experienced LH strokes, only LH ROIs were analyzed. To reduce false positives, Freedman–Lane correction (Faria, 2012) was applied using 2000 permutation tests (this was found to yield the same results as 10,000 permutations, but requires less computational resources, making for more efficient analyses), with age and regional cerebellar GMV as covariates, and statistical significance set at p-value < 0.05. For interpretability, we define “strong associations” as those with z-values ≤ –3.0, “moderate” as –3.0 < z ≤ –2.8 (still significant), and note that all reported associations met permutation-corrected thresholds at p < 0.05.

### 2.5 Additional lesion symptom mapping analysis – apraxia of speech

To explore whether the lesion patterns associated with cerebellar degeneration overlapped with regions affected in speech-motor impairments, we conducted a separate LSM analysis using AOS Total score from the Apraxia of Speech Rating Scale (ASRS) (Winkler et al., 2014) as the predicted variable. Mirroring our cerebellar LSM, these analyses were conducted using the NiiStat toolbox, including age at scan as a covariate and applying Freedman–Lane permutation testing (2000 permutations) with a statistical significance threshold of p < 0.05, permutation-corrected. Lesion maps were compared across 85 LH ROIs derived from the combined JHU grey and white matter atlas (Strand et al., 2014). This approach allowed us to assess whether damage to the same left hemisphere structures that predicted cerebellar gray matter atrophy also contributed to behavioral speech-motor impairment, and whether cerebellar-related tracts play a dual role in structural and functional outcomes following stroke.

## 3 Results

**Fig. 2:**
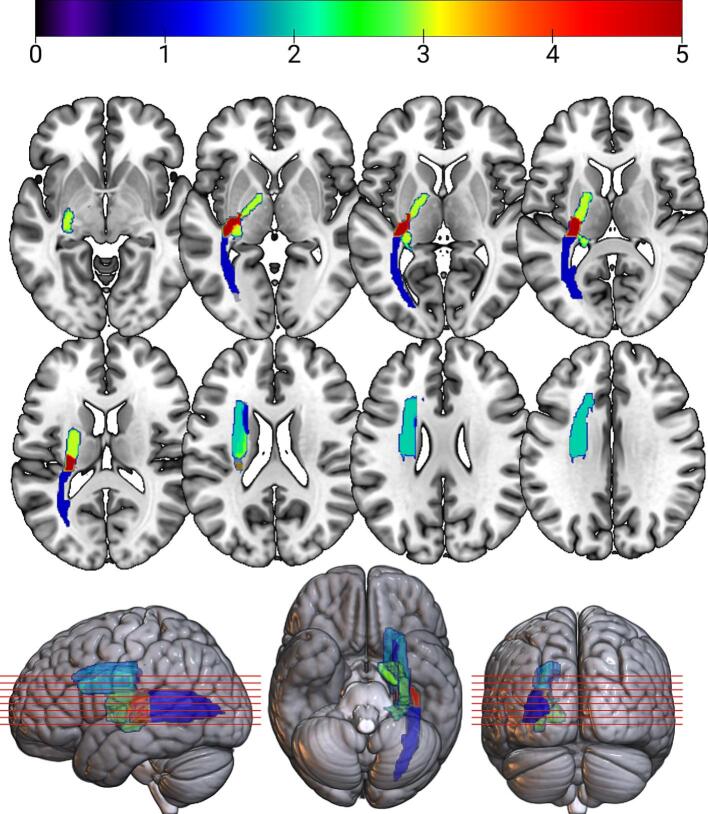
An overlay map shown in neurological convention of JHU atlas–based ROIs identified through LSM, where each included region showed a significant negative association (Z < –2.8) with gray matter volume in a particular cerebellar ROI. Each ROI’s intensity indicates the number of distinct cerebellar ROIs for which that region was found to be significantly associated, with values ranging from 0 (no association) to 5 (red, lesioned areas associated with 5 cerebellar ROIs). The analysis revealed consistent lesion overlap across structures within the left PLIC, Fx/ST, and RLIC. Axial slices and 3D renderings are shown on the SPM152 template to visualize the spatial distribution of supratentorial regions most strongly linked to regional cerebellar GMV in this chronic stroke cohort (MNI coordinate space at z-dimension intervals of −5, 0, 5, 10, 15, 20, 25, and 30). (For interpretation of the references to color in this figure legend, the reader is referred to the web version of this article.)

**Fig. 3:**
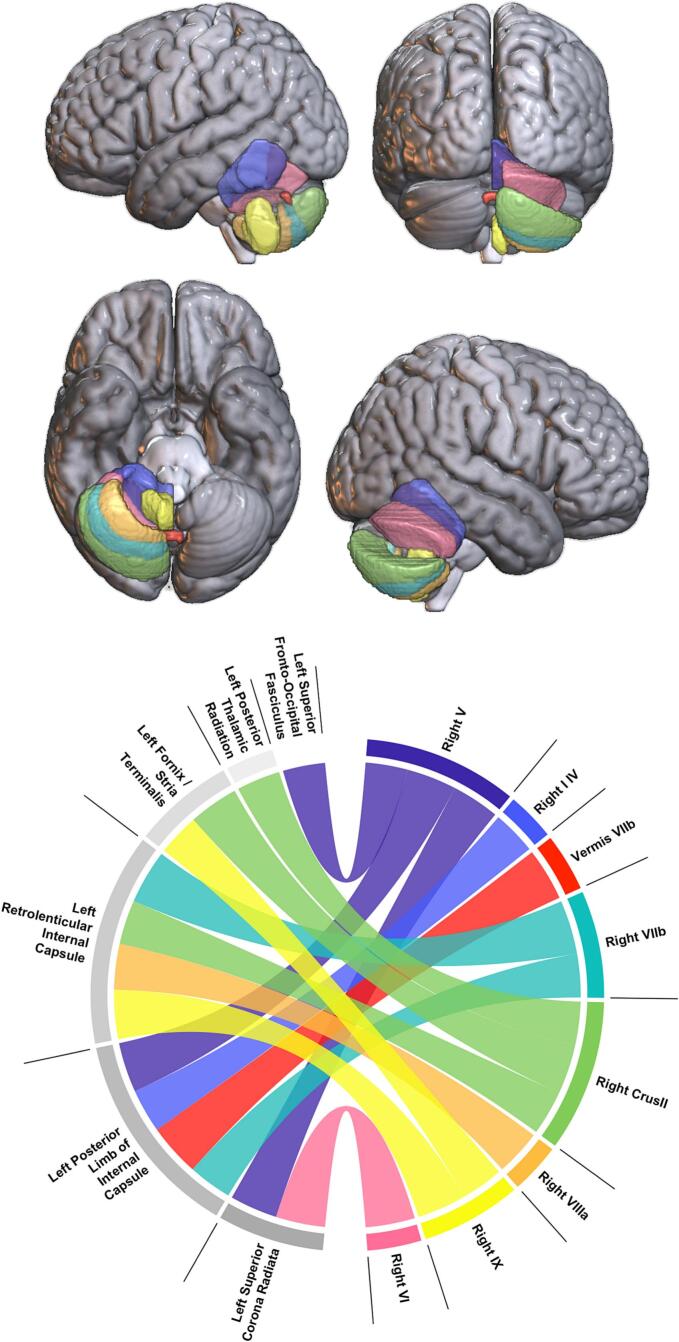
Chord diagram illustrating cortical lesion areas associated with cerebellar GMV ROIs. Colors are labeled to cerebellar ROIs as follows: Pink = Right VI, Yellow = Right IX, Orange = Right VIIIa, Green = Right Crus II, Teal = Right VIIb, Red = Vermis VIIb, Blue = Right I-IV, Violet = Right V. (For interpretation of the references to color in this figure legend, the reader is referred to the web version of this article.)

VLSM and ROI analyses revealed significant associations between lesion location and cerebellar GMV degradation across multiple regions in chronic left-hemisphere stroke survivors *(*Table 1*,* Fig. 2*)*. Notably, lesions involving the left posterior limb of the internal capsule (PLIC), the superior corona radiata (SCR), the retrolenticular part of the internal capsule (RLIC), and the fornix/stria terminalis (Fx/ST) were associated with the most robust effects on cerebellar gray matter volume (z-values ≤ –3.0), which we define as “strong” associations throughout the manuscript (Fig. 3).

**Table 1:** Cortical lesion sites associated with cerebellar regional GMV integrity.

**ROIs Associated with Cerebellar GMV**	**# of Associated Cerebellar ROIs**	**Cerebellar ROI: Z-value**
Retrolenticular Part of the Internal Capsule (RLIC)	5	Right Crus II: −3.108 Vermis VIIb: −3.669 Right VIIb: −3.768 Right VIIIa: −3.202 Right IX: −3.434
Fornix (cres) / Stria Terminalis (Fx/ST)	3	Right IX: −2.946 Right VIIb: −3.099 Right Crus II: −3.471
Posterior Limb of the Internal Capsule (PLIC)	3	Right I-IV: −3.066 Right V: −3.482 Right VIIb: −3.046
Superior Corona Radiata (SCR)	2	Right V: −3.331 Right VI: −3.445
Posterior Thalamic Radiation (PTR)	1	Right Crus II: −3.046
Superior Fronto-Occipital Fasciculus (SFO)	1	Right V: −2.949

Lesions in the left PLIC (z = −3.07) were associated with reduced GMV in the right lobules I-IV, while the left SCR (z = −3.33), PLIC (z = −3.48) and superior fronto-occipital fasciculus (SFO; z = −2.95) lesions predicted atrophy in the right lobule V (Fig. 2). Further, degeneration in right lobule VI was associated with lesions in the left SCR (z = −3.44). The right Crus II showed associations with lesions in the left RLIC (z = −3.11), Fx/ST (z = −3.47), and posterior thalamic radiation (PTR, z = −3.05).

Cerebellar GMV reductions in vermis VIIb were predicted by lesions in the left RLIC (z = −3.67), while the right lobule VIIb was linked to lesions in the left PLIC (z = −3.05), RLIC (z = −3.77), and Fx/ST (z = −3.10). Additionally, atrophy in the right lobule VIIIa was associated with lesions in the left RLIC (z = −3.20), and reductions in the right lobule IX were linked to lesions in the left RLIC (z = −3.43) and Fx/ST (z = −2.95). To visualize the cumulative impact of these associations, an overlay map was generated by averaging Z-values across all significant ROIs (Fig. 3), highlighting regions where lesion location was most strongly associated with cerebellar degeneration.

No associations were found between lesion location and GMV degradation in the left cerebellar lobules or the vermis in most regions (Vermis VIIb was the only region outside of the right lobule to produce significant results, z = −3.67). The results indicate that lesion location, particularly involving key white matter tracts, is a predictor of cerebellar atrophy.

Additionally, analyses including time post-stroke as a predictor revealed no significant associations with cerebellar GMV across any regions, nor did controlling for time post-stroke alter the main lesion-symptom mapping findings. These null results suggest that chronic cerebellar degeneration in this cohort is not solely dependent on time since stroke.

### 3.1 Predictors of apraxia of Speech, and overlap with predicting cerebellar volume

Voxel-based lesion-symptom mapping using AOS Total score revealed significant associations with 10 left-hemisphere regions, spanning cortical, subcortical, and white matter structures. Specifically, higher AOS severity was predicted by lesions to the left inferior frontal gyrus pars opercularis (z = 4.31), precentral gyrus (z = 5.39), postcentral gyrus (z = 4.63), and supramarginal gyrus (z = 4.26), consistent with known speech-motor control regions. Additional significant associations were found in the left insula (z = 3.48) and posterior insula (z = 4.39), as well as white matter pathways including the superior longitudinal fasciculus (z = 5.55), external capsule (z = 4.02), superior corona radiata (z = 4.17), and retrolenticular part of the internal capsule (z = 3.58).

Importantly, two of these regions, the left SCR and the RLIC, were also previously identified as predictors of cerebellar GMV reductions. Specifically, lesions to left SCR were associated with GMV loss in right lobules V (z = –3.33) and VI (z = –3.45), and lesions to the left RLIC were linked to GMV reductions in right Crus II (z = –3.11), VIIb (z = –3.77), VIIIa (z = –3.20), IX (z = –3.43), and vermis VIIb (z = –3.67). These overlapping lesion predictors suggest that disruption to specific white matter tracts may contribute both to remote cerebellar degeneration and to speech-motor impairment, potentially reflecting a shared vulnerability along the cortico-ponto-cerebellar and dorsal stream pathways.

## 4 Discussion

The overarching goal of this study was to investigate the relationship between the location of cortical stroke lesions and cerebellar atrophy in a cohort of chronic left hemisphere stroke patients. Contrary to the common assumption that post-stroke cerebellar degeneration is primarily a function of global lesion severity, we hypothesized that specific lesion locations are a stronger determinant of cerebellar GMV loss. In particular, we proposed that lesions affecting key components of the cortico-cerebellar pathways, especially areas such as the PLIC and SCR, would show the strongest associations with cerebellar degeneration. Our findings support this hypothesis, demonstrating strong associations (defined as z-scores ≤ –3.0) between lesions in these regions and GMV loss in the contralesional cerebellar lobules and vermis.

Overall, GMV in the left cerebellar lobules and vermis showed no significant associations with any cortical lesion ROIs, suggesting that cerebellar degeneration following stroke is not due to direct injury but rather a secondary effect of contralateral cortical and subcortical lesions. This finding aligns with the concept of crossed cerebellar diaschisis,^39^ where damage to the cerebral hemisphere results in reduced metabolism and blood flow in the opposite cerebellar regions. While our analysis included cortical speech ROIs (e.g., inferior frontal gyrus, precentral and postcentral gyri), none were significantly associated with cerebellar GMV reductions. This suggests that lesion location along major white matter pathways, rather than isolated damage to cortical speech areas, may play a more dominant role in post-stroke cerebellar degeneration.

Our results emphasize the specificity of lesion distribution, beyond total volume, in explaining cerebellar atrophy. While the findings are observational and cross-sectional, they offer a structural framework that may inform future studies on how specific pathways contribute to remote degeneration. Although this highlights the call to consider lesion location in stroke rehabilitation planning, especially for patients with motor speech deficits, any clinical application of these results would require prospective validation. Identifying specific regions of the cortico-cerebellar pathways that are most predictive of cerebellar neurodegeneration may help identify patients at risk for long-term cerebellar degeneration and guide targeted rehabilitation strategies. Ultimately, by improving our understanding of the anatomical and functional connections between the cerebrum and cerebellum, we can improve predictive models and address the functional deficits experienced by stroke survivors, and particularly those related to motor speech control.

### 4.1 Shared lesion location of cerebellar atrophy & motor-speech impairment

While AOS severity was associated with lesions across 10 left-hemisphere regions, including expected speech-motor areas such as the inferior frontal gyrus (z = 4.31) and precentral gyrus (z = 5.39), only two white matter regions emerged as predictors of both AOS severity and cerebellar atrophy. Lesions to the left SCR (z = 4.17) were associated with both motor-speech impairment and less GMV in right cerebellar lobules V (z = −3.33) and VI (z = −3.45). Similarly, damage to the left RLIC (z = 3.58) predicted AOS severity alongside widespread cerebellar atrophy, relating to the right Crus II (z = −3.11), VIIb (z = −3.77), VIIIa (z = -3.20), IX (z = −3.43), and vermis VIIb (z = −3.67). While z-score directions differed due to variable scaling (lower cerebellar GMV versus higher AOS severity), both reflected negative lesion effects, reinforcing these tracts' shared role in structural and functional outcomes.

These white matter regions likely function as critical relay points, transporting signals from the cortex to brainstem nuclei that project to cerebellum. Damage here may simultaneously disrupt speech-motor control and cerebellar connectivity, explaining the dual vulnerability. This suggests that SCR and RLIC lesions may predict both chronic AOS and progressive cerebellar changes, supporting a network-based view of post-stroke outcomes.

### 4.2 Limitations

One of our primary limitations include the cross-sectional design, which prevents tracking the longitudinal progression of cerebellar degeneration. Longitudinal research could provide a temporal understanding of how cerebellar atrophy develops after stroke, particularly in relation to lesion location and neural adaptations.

Furthermore, the focus on chronic left-hemisphere stroke survivors restricts the generalizability of the findings. Future research should include right-hemisphere stroke patients to explore potential differences in cerebellar atrophy patterns, considering the brain's lateralization and the potential for distinct degeneration mechanisms across hemispheres.

## 5 Conclusion

These findings provide evidence of remote structural consequences following stroke while emphasizing the importance of lesion location in predicting cerebellar degeneration and potentially informing rehabilitation strategies. In this study, we investigated the relationship between lesion location and cerebellar GMV degradation in a chronic left-hemisphere stroke cohort, with our findings highlighting the critical role of specific white matter tracts, particularly the PLIC, SCR, RLIC, and Fx/ST, in predicting cerebellar atrophy. Based on this we can infer that cerebellar degeneration is not random, but a result of disconnection caused by an insult to specific areas. These regions are major components of the cortico-cerebellar pathways that facilitate motor and speech functions, and damage to these pathways appears to trigger significant cerebellar degeneration through, what we can assume is, CCD.

Our VLSM and region-based analyses revealed several key associations between specific locations of cortical lesions and cerebellar atrophy. For example, lesions within the left PLIC and SCR were strongly linked to atrophy in solely the right cerebellar lobules, including lobules I-IV, V, and VI. The SFO also emerged as a significant region associated with degeneration only in right lobule V, adding to the growing recognition of its role in transhemispheric cortico-cerebellar circuits. Furthermore, lesions within the left RLIC and Fx/ST were associated with atrophy in the right Crus II, lobules VIIb, VIIIa, and IX. Notably, the right vermis VIIb showed significant GMV reductions due to lesions in the left RLIC, further suggesting the disruption of critical cortico-cerebellar connections.

Given this contribution to our understanding of remote degeneration following cortical stroke, these findings provide a foundation for future longitudinal studies. The present results do not imply prediction of future degeneration but rather offer a structural framework for hypothesis generation in future research on diaschisis and neural resilience. Cerebellar grey matter volume is not decreasing in all cortical stroke individuals, and we do not see this across all regions. Therefore, future investigations can make informed assumptions regarding prognosis, and improved approaches.

Finally, our findings offer a potential framework for assessing cerebellar preservation relative to lesion burden. Mapping lesion-atrophy relationships may help clinicians identify individuals whose cerebellar structure appears preserved beyond what would be expected, serving as a potential marker of neural integrity and capacity for functional recovery. In addition, exploratory analyses of apraxia of speech severity revealed overlapping lesion predictors with those associated with cerebellar atrophy, providing further support that disruption along cortico-cerebellar pathways contributes to both structural and functional outcomes. This approach could ultimately aid in tailoring treatment strategies to individuals who may be more resilient based solely on anatomy.

During the preparation of this work the authors used ChatGPT in order to improve flow and clarity. After using this tool/service, the authors reviewed and edited the content as needed and take full responsibility for the content of the publication.

## Ethical considerations

All participants gave informed consent for study participation in accordance with the Declaration of Helsinki.

## CRediT authorship contribution statement

**Makayla Gibson:** Writing – review & editing, Writing – original draft, Visualization, Validation, Methodology, Investigation, Formal analysis, Conceptualization. **Roger Newman-Norlund:** Writing – review & editing, Visualization, Supervision, Software, Resources. **Janina Wilmskoetter:** Writing – review & editing. **Sigfus Kristinsson:** Writing – review & editing, Resources. **Leonardo Bonilha:** Writing – review & editing, Funding acquisition. **Julius Fridriksson:** Writing – review & editing, Funding acquisition. **Chris Rorden:** Writing – review & editing, Visualization, Supervision, Software, Resources, Funding acquisition.

## Funding

This work was supported by the National Institute of Health (P50DC014664, U01DC011739, R01DC008355, RF1MH133701). We would like to acknowledge the participants, students, faculty and staff who have supported the Center for the Study of Aphasia Recovery.

## Declaration of competing interest

The authors declare that they have no known competing financial interests or personal relationships that could have appeared to influence the work reported in this paper.

## Data Availability

A partial dataset can be found on OpenNeuro (Aphasia Recovery Cohort; https://openneuro.org/datasets/ds004884/versions/1.0.2). This is cited in our Methods section.
